# Optimizing predictive features using machine learning for early miscarriage risk following single vitrified-warmed blastocyst transfer

**DOI:** 10.3389/fendo.2025.1557667

**Published:** 2025-04-16

**Authors:** Lidan Liu, Bo Liu, Huimei Wu, Qiuying Gan, Qianyi Huang, Mujun Li

**Affiliations:** ^1^ Guangxi Reproductive Medical Center, The First Affiliated Hospital of Guangxi Medical University, Nanning, China; ^2^ Reproductive Center, Nanning Maternity and Child Health Hospital, Nanning, Guangxi, China

**Keywords:** early miscarriage, single vitrified-warmed blastocyst transfer (SVBT), machine learning (ML), voting classifier, gradient boosting

## Abstract

**Research question:**

Can machine learning models accurately predict the risk of early miscarriage following single vitrified-warmed blastocyst transfer (SVBT)?

**Design:**

A dual-center retrospective analysis of 1,664 SVBT cycles, including 308 early miscarriage cases, was conducted across two reproductive centers. Multiple machine learning models, such as Logistic Regression, Random Forest, Gradient Boosting, and Voting Classifier, were developed. Metrics including Area Under the Curve(AUC), accuracy, precision, recall, F1 score, and specificity were used to evaluate model performance. Key predictors were identified through Mutual Information and Recursive Feature Elimination (RFE).

**Results:**

Maternal age, paternal age, endometrial thickness, blastocyst quality, and ovarian stimulation parameters were identified as critical predictors. Compared to traditional statistical models such as logistic regression (AUC = 0.584), ensemble models demonstrated significantly improved predictive performance. The Voting Classifier achieved the highest AUC (0.836), accuracy (0.780), precision (0.914), and specificity (0.942), outperforming individual machine learning classifiers. The Gradient Boosting Classifier also exhibited strong performance (AUC 0.831, accuracy 0.777), confirming the effectiveness of ensemble learning in capturing complex predictors of early miscarriage risk.

**Conclusion:**

Ensemble machine learning models, particularly the Voting Classifier and Gradient Boosting Classifier, significantly improve the prediction of early miscarriage following SVBT. These models provide accurate, individualized risk assessments, enhancing clinical decision-making and advancing personalized care in ART.

## Introduction

Early miscarriage, defined as the spontaneous loss of pregnancy before 12 weeks and 6 days of gestation, is a common complication in *in vitro* fertilization (IVF) pregnancies, affecting 10-15% of cases, with 80% occurring in the first trimester (1, 2). In single vitrified-warmed blastocyst transfer (SVBT), early miscarriage risk remains significant due to the multifactorial nature of its causes, including genetic abnormalities, uterine factors, hormonal imbalances, and environmental influences (1, 3, 4). Accurate predictive models are essential for identifying at-risk pregnancies and optimizing clinical decision-making.

Machine learning (ML) offers a powerful approach for early miscarriage risk prediction, as it can process large, heterogeneous datasets and capture complex, non-linear relationships between multiple clinical, embryological, and demographic variables (5, 6). Traditional statistical models, such as logistic regression and LASSO regression, have been widely applied in reproductive medicine but have demonstrated only moderate predictive performance, with AUC values ranging from 0.615 to 0.660 (6).These models often assume linear relationships among predictors and may struggle with the intricate interdependencies inherent in reproductive outcomes. In contrast, ML techniques, particularly ensemble learning methods, can leverage multiple algorithms to improve predictive accuracy, robustness, and generalizability (7).

Ensemble learning, which combines the predictions of multiple models, has demonstrated significant advantages in improving predictive accuracy and robustness. Common techniques include Voting, Stacking, and Boosting. Voting aggregates predictions from several models to produce a final result, Stacking uses the outputs of multiple models as inputs for a meta-model, and Boosting builds models sequentially, with each iteration correcting errors from the previous one (7). Ensemble methods have been successfully applied in various medical domains, such as cancer risk prediction, where Stacking models improved the classification of tumor types (8), and in diagnosing cardiovascular diseases, where Boosting algorithms enhanced early detection of heart conditions (9). These successes underscore ensemble learning’s ability to integrate diverse features, reduce bias and variance, and improve stability when applied to complex clinical datasets. Given the multifactorial nature of early miscarriage, ensemble methods are particularly suited for capturing the intricate relationships between predictive factors and delivering reliable predictions (10, 11).

SVBT is widely used in assisted reproductive technologies (ART) due to its ability to reduce the risks associated with multiple pregnancies, such as preterm birth and low birth weight. Despite its advantages, including improved timing flexibility and enhanced endometrial synchronization (12), the risk of early miscarriage following SVBT remains a concern. Recent advancements in ML have demonstrated its efficacy in medical diagnosis and risk assessment, including applications in obstetrics and gynecology. For example, ML models have been used to predict pregnancy complications and implantation failure (13–15).

To date, no predictive models have been specifically developed for early miscarriage in SVBT cycles. This study aims to develop and evaluate machine learning models to predict early miscarriage risk following SVBT by conducting a comparative analysis of multiple approaches, including Logistic Regression, Random Forest, Gradient Boosting, and ensemble methods, to identify the most effective predictive model. Additionally, we integrate ensemble learning techniques, specifically the Voting Classifier and Stacking Classifier, to leverage the strengths of multiple models, further enhancing predictive accuracy and supporting more informed clinical decision-making.

## Materials and methods

### Study design and population

**Figure 1:**
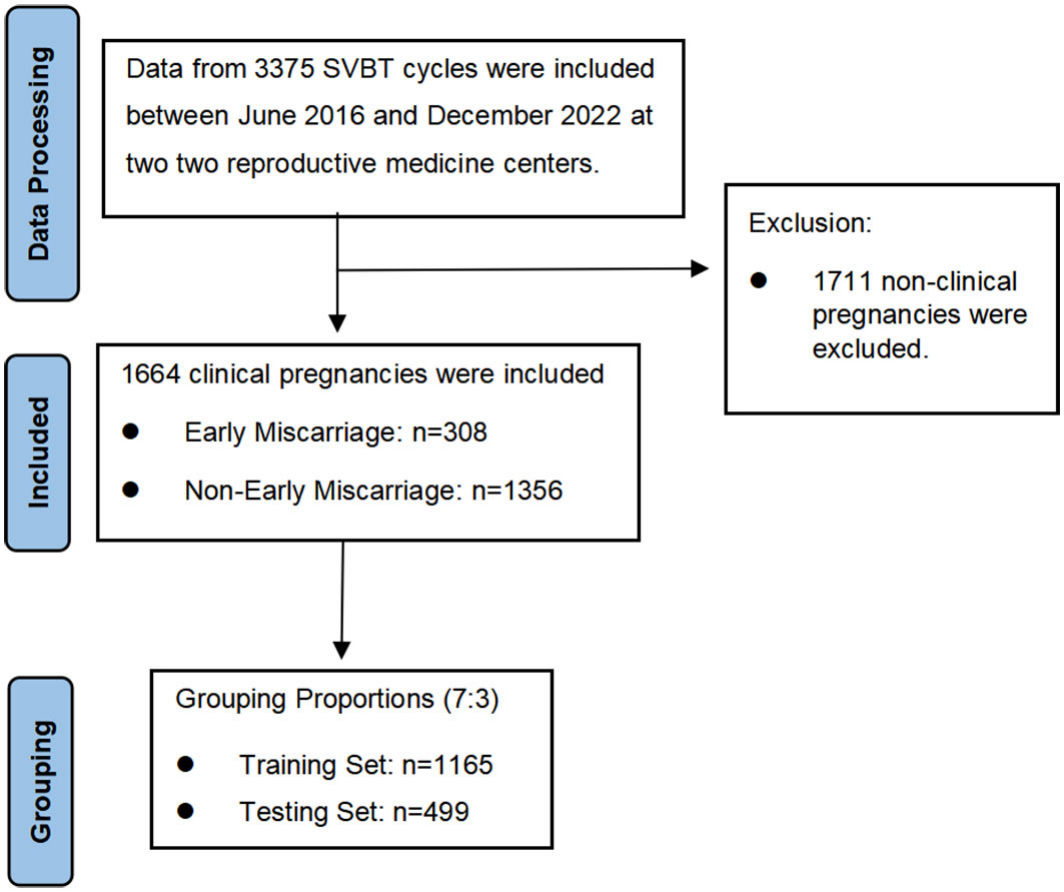
Flowchart illustrating the selection process of participants for this study.

This retrospective study was conducted at two reproductive medicine centers: the First Affiliated Hospital of Guangxi Medical University and the Nanning Maternity and Child Health Hospital. A total of 3,375 SVBT cycles performed between June 2016 and December 2022 were reviewed, among which 1,664 resulted in clinical pregnancies and 308 ended in early miscarriage. To ensure robust model development, data were randomly divided into training (70%) and testing (30%) sets. The inclusion criteria were patients undergoing SVBT, with complete clinical and laboratory records. **Figure 1** outlines the study flowchart. Both centers adhered to identical laboratory and clinical protocols, ensuring consistency in patient preparation, embryo culture, and transfer procedures.

### Data collection and validation process

Clinical and laboratory data were extracted from the EMRs of the participating centers. To ensure data accuracy, two independent clinical data managers validated key variables, including patient demographics, clinical protocols, and pregnancy outcomes. Any inconsistencies were cross-checked with original medical records before inclusion in the study. Model performance was evaluated using repeated stratified 10-fold cross-validation, ensuring proportional representation of early miscarriage cases across folds. This rigorous approach minimizes overfitting, enhances model reliability, and ensures reproducibility of findings.

### Ovarian stimulation and oocyte insemination

Ovarian stimulation protocols were tailored to individual patients based on clinical parameters such as age, BMI, baseline FSH levels, and antral follicle counts (16). Triggering of ovulation was achieved with human chorionic gonadotropin (HCG) when at least one follicle reached 18 mm in diameter, and oocyte retrieval was performed 36 hours later under ultrasound guidance. Insemination, either through conventional IVF or ICSI, was determined based on semen quality, following standard protocols at the participating centers.

### Embryo culture and blastocyst scoring

The blastocysts were cultured continuously in a single culture medium throughout all developmental stages and incubated under oil at 37°C in an environment containing 5% O2 and 6% CO2, with nitrogen as the balance gas. Blastocyst assessments were conducted using the Gardner scoring system (17).

### Blastocyst vitrification and thawing procedures

Fully expanded blastocysts were artificially shrunk using a laser before being cryopreserved with vitrification kits (KITAZATO). The embryos were then loaded onto a cryotop on day 5-6 post-insemination. The cryopreserved blastocysts were stored in liquid nitrogen until they were ready to be warmed. Blastocyst warming was performed using warming kits (KITAZATO) once the endometrium achieved adequate thickness. The survival of the blastocyst was assessed by its re-expansion two hours post-warming.

### Endometrial preparation and blastocyst transfer

Endometrial preparation for frozen embryo transfer (FET) followed four main protocols:

1. Modified Natural Cycle (NC): Ovulation was induced with HCG when the dominant follicle reached ≥18 mm, followed by luteal support with dydrogesterone or vaginal progesterone. Blastocyst transfer was performed 5 days post-ovulation.
2. Mild Stimulation Cycle (MS): For cases with insufficient follicular development, human menopausal gonadotropin (HMG) was administered to stimulate follicular growth. Ovulation was triggered with HCG, and luteal support was initiated before transfer.
3. Hormone Replacement Therapy (HRT): Endometrial preparation included estradiol valerate for endometrial proliferation, followed by progesterone for luteal support. Patients received either intramuscular or vaginal progesterone, combined with dydrogesterone, based on clinical needs.
4. GnRH Agonist Combined with HRT (GnRHa-HRT): Downregulation was achieved using triptorelin acetate (GnRH agonist) administered during the early follicular phase. Hormonal and endometrial parameters were monitored until complete downregulation was confirmed (e.g., estradiol <50 pg/mL, FSH <5 IU/L, LH <5 IU/L, endometrial thickness <5 mm). Following downregulation, estradiol valerate and progesterone were used to prepare the endometrium, with blastocyst transfer performed 6 days after initiating progesterone.

All blastocyst transfers were conducted under abdominal ultrasound guidance (18). Protocol selection was individualized based on patient characteristics and clinical indications.

### Clinical outcomes

The primary outcome was early miscarriage, defined as the spontaneous loss of a pregnancy before 12 weeks and 6 days of gestation.

### Data acquisition and potential predictors

The initial dataset comprised more than 40 features, capturing a broad range of maternal, paternal, embryonic, and clinical characteristics relevant to early miscarriage risk. A panel of three reproductive medicine experts guided the feature selection process based on clinical relevance and literature review, ultimately selecting 32 features for model development. The selected features include: maternal age, paternal age, body mass index (BMI), basal FSH, previous gravidity, infertility duration, Gonadotropin (Gn) duration, total Gn dose, number of oocytes retrieved, endometrial thickness, basal LH, trigger day estradiol, blastulation time, blastocyst stage, inner cell mass (ICM), trophectoderm (TE), cleavage stage fragmentation, number of blastomeres at the cleavage stage, infertility type, previous parity, previous abortus, number of previous transfers, infertility cause, controlled ovarian hyperstimulation (COH) protocol, fertilization method, and endometrial preparation.

### Data preparation and equilibration

This research included a total of 1,664 cycles, of which 1,356 did not result in early miscarriage and 308 did. The dataset was complete with no missing values, encompassing data from individuals who underwent single vitrified-warmed blastocyst transfers. To ensure compatibility with machine learning algorithms, categorical variables (e.g., endometrial preparation protocol) were encoded using Label Encoding, while continuous variables (e.g., maternal age, BMI, endometrial thickness) were standardized using Min-Max Scaling to enhance model convergence and comparability.

The dataset was split using train_test_split, with 70% allocated to the training set and 30% to the testing set. The stratify=y parameter was applied to ensure stratified sampling, maintaining the class distribution consistency. Given the significant class imbalance, with more cases of non-early miscarriage than early miscarriage, we employed the SMOTETomek technique (19). This method combines SMOTE (Synthetic Minority Over-sampling Technique) and Tomek Links to balance the classes effectively. First, SMOTE generates synthetic samples for the minority class (early miscarriage) to increase its representation in the dataset, helping to prevent the model from becoming biased towards the majority class (non-miscarriage) during training. Next, Tomek Links refine the dataset by identifying and removing overlapping samples that are difficult to classify, enhancing the clarity of the decision boundary between the classes.

### Feature optimization

To determine the most pertinent features for predicting early miscarriage, we used Mutual Information (MI), a statistical metric that measures the dependency between two random variables. MI quantifies how much knowing one feature reduces the uncertainty of the other, capturing all possible relationships between the features, not just linear ones. A higher mutual information value indicates a stronger dependency between the features.

Recursive Feature Elimination (RFE) combined with a Random Forest classifier was employed as a feature selection technique to identify the most important features, enhancing both the predictive performance and interpretability of the model. RFE works by recursively training the model and removing the least important features in each iteration, gradually reducing the feature set size until the desired number of features is reached. In each iteration, feature importance is computed by the Random Forest classifier, which effectively captures complex nonlinear relationships between features.

Based on the results from MI and RFE analyses, we selected an optimal number of features for inclusion in our model. This approach ensured that the chosen features demonstrated strong relationships and significant overall dependencies with the target variable, early miscarriage, thereby optimizing the model’s predictive power and interpretability.

### Machine learning methodology and assessment

Eight machine learning classifiers were evaluated for their ability to predict early miscarriage, including Logistic Regression, Random Forest, Gradient Boosting, and CatBoost. Models were trained using a balanced dataset (SMOTETomek technique) to address class imbalance. Performance was assessed using repeated stratified 10-fold cross-validation, with AUC, accuracy, recall, precision, F1 score, and specificity as key metrics. Ensemble methods, including Voting and Stacking Classifiers, were constructed using top-performing models to further enhance predictive accuracy.

### Ensemble model construction and evaluation

To enhance the performance of early miscarriage prediction models, this study employed ensemble learning methods by constructing two ensemble models: a Voting Classifier and a Stacking Classifier. First, the two best-performing models were selected as base models to construct the Voting Classifier, utilizing a soft voting strategy based on predicted probabilities. The Stacking Classifier combined these two base models, with Logistic Regression serving as the meta-classifier. Both ensemble models were trained on the training data and subsequently used to make predictions and evaluations on the test data. The evaluation metrics included AUC, accuracy, recall, precision, F1 score, and specificity.

### Statistical analysis

Statistical analysis was conducted using Python software (Version 3.12). Participant characteristics were summarized using means and standard deviations for continuous variables, and frequencies and percentages for categorical variables. T-tests were employed to compare differences between continuous variables, while chi-square tests or Fisher’s exact tests were used for categorical variables. This approach ensured a robust and accurate assessment of the data.

## Results

### Baseline characteristics of SVBT cycles stratified by early miscarriage outcomes

Of the 1,664 SVBT cycles analyzed, significant differences were observed between the miscarriage and non-miscarriage groups. Advanced maternal and paternal ages were associated with higher miscarriage risk, with median ages of 33 and 35 years compared to 32 and 33 years in the non-miscarriage group (p<0.001). The miscarriage group also exhibited thinner endometrial thickness (9.3 mm vs. 9.5 mm, p=0.031), delayed blastocyst development by day 5 (57.5% vs. 64.9%, p=0.017), and poorer inner cell mass (ICM) quality (grade C: 14.0% vs. 8.4%, p<0.01). Additionally, ovarian-related infertility was more prevalent in the miscarriage group (30.8% vs. 21.5%, p=0.001). These findings highlight the multifactorial nature of miscarriage risk, emphasizing the importance of integrating parental, embryonic, and endometrial factors into predictive models (**Table 1**).

**Table 1:** Baseline characteristics of 1664 SVBT cycles stratified by early miscarriage outcomes.

Variable	Overall (N=1664)	Early Miscarriage (N=308)	Non-Early Miscarriage (N=1356)	p value
Maternal age (median [IQR])	32.0 [29.0, 35.0]	33.0 [30.0, 37.0]	32.0 [29.0, 35.0]	<0.001***
Paternal age (median [IQR])	34.0 [31.0, 37.0]	35.0 [31.0, 39.0]	33.0 [30.0, 37.0]	<0.001***
Maternal BMI (median [IQR])	21.2 [19.6, 23.3]	21.5 [19.9, 23.1]	21.2 [19.6, 23.4]	0.538
Basal FSH (median [IQR])	6.1 [5.3, 7.1]	6.2 [5.3, 7.1]	6.1 [5.3, 7.1]	0.484
Basal LH (median [IQR])	5.5 [4.2, 7.3]	5.4 [4.3, 7.0]	5.5 [4.2, 7.3]	0.926
Infertility duration (median [IQR])	3.6 [2.0, 5.8]	4.0 [2.0, 6.0]	3.4 [2.0, 5.5]	0.181
Trigger day estradiol (median [IQR])	4672.0 [3087.5, 6397.0]	4446.0 [2953.2, 6313.0]	4697.4 [3144.0, 6407.2]	0.112
Gn duration (median [IQR])	11.0 [10.0, 12.0]	11.0 [9.8, 12.0]	11.0 [10.0, 12.0]	0.993
Total Gn dose (median [IQR])	1950.0 [1500.0, 2681.2]	2025.0 [1500.0, 2850.0]	1925.0 [1462.5, 2625.0]	0.177
Number of oocytes retrieved (median [IQR])	20.0 [16.0, 26.0]	20.0 [14.0, 26.0]	20.0 [16.0, 26.0]	0.181
Endometrial thickness (median [IQR])	9.5 [8.5, 10.7]	9.3 [8.2, 10.3]	9.5 [8.5, 10.8]	0.031*
Blastulation time (%)				
Day 5	1057 (63.5)	177 (57.5)	880 (64.9)	0.017*
Day 6	607 (36.5)	131 (42.5)	476 (35.1)	
Blastocyst stage (%)				
3	300 (18.0)	68 (22.1)	232 (17.1)	0.089
4	996 (59.9)	180 (58.4)	816 (60.2)	
5	336 (20.2)	52 (16.9)	284 (20.9)	
6	32 (1.9)	8 (2.6)	24 (1.8)	
ICM (%)				
A	694 (41.7)	115 (37.3)	579 (42.7)	0.007**
B	813 (48.9)	150 (48.7)	663 (48.9)	
C	157 (9.4)	43 (14.0)	114 (8.4)	
TE (%)				
A	758 (45.6)	131 (42.5)	627 (46.2)	0.452
B	787 (47.3)	152 (49.4)	635 (46.8)	
C	119 (7.2)	25 (8.1)	94 (6.9)	
Cleavage stage fragmentation (%)				
≤10%	817 (49.1)	150 (48.7)	667 (49.2)	0.814
11%-25%	787 (47.3)	145 (47.1)	642 (47.3)	
26%-50%	60 (3.6)	13 (4.2)	47 (3.5)	
Number of blastomeres at the cleavage stage (%)				
4	144 (8.7)	33 (10.7)	111 (8.2)	0.002**
5	161 (9.7)	47 (15.3)	114 (8.4)	
6	330 (19.8)	65 (21.1)	265 (19.5)	
7	260 (15.6)	37 (12.0)	223 (16.4)	
8	709 (42.6)	116 (37.7)	593 (43.7)	
9	34 (2.0)	7 (2.3)	27 (2.0)	
≥10	26 (1.6)	3 (1.0)	23 (1.7)	
Infertility type				
PI	686 (41.2)	132 (42.9)	554 (40.9)	0.562
SI	978 (58.8)	176 (57.1)	802 (59.1)	
Previous gravidity (%)				
0	686 (41.2)	132 (42.9)	554 (40.9)	0.63
1	501 (30.1)	92 (29.9)	409 (30.2)	
2	265 (15.9)	42 (13.6)	223 (16.4)	
≥3	212 (12.7)	42 (13.6)	170 (12.5)	
Previous parity (%)				
0	1250 (75.1)	218 (70.8)	1032 (76.1)	0.041*
1	384 (23.1)	80 (26.0)	304 (22.4)	
2	28 (1.7)	10 (3.2)	18 (1.3)	
3	2 (0.1)	0 (0.0)	2 (0.1)	
Previous abortus (%)				
0	1024 (61.5)	198 (64.3)	826 (60.9)	0.084
1	475 (28.5)	73 (23.7)	402 (29.6)	
2	123 (7.4)	25 (8.1)	98 (7.2)	
>=3	42 (2.5)	12 (3.9)	30 (2.2)	
Number of previous transfers (%)				
0	818 (49.2)	146 (47.4)	672 (49.6)	0.765
1	550 (33.1)	101 (32.8)	449 (33.1)	
2	186 (11.2)	39 (12.7)	147 (10.8)	
≥3	110 (6.6)	22 (7.1)	88 (6.5)	
Infertility cause				
Tubal (%)				
NO	383 (23.0)	73 (23.7)	310 (22.9)	0.809
YES	1281 (77.0)	235 (76.3)	1046 (77.1)	
Endometriosis (%)				
NO	1568 (94.2)	281 (91.2)	1287 (94.9)	0.018
YES	96 (5.8)	27 (8.8)	69 (5.1)	
Unexplained (%)				
NO	1513 (90.9)	281 (91.2)	1232 (90.9)	0.921
YES	151 (9.1)	27 (8.8)	124 (9.1)	
Male factor (%)				
NO	1138 (68.4)	214 (69.5)	924 (68.1)	0.698
YES	526 (31.6)	94 (30.5)	432 (31.9)	
Uterine_causes (%)				
NO	1598 (96.0)	298 (96.8)	1300 (95.9)	0.579
YES	66 (4.0)	10 (3.2)	56 (4.1)	
Ovarian_causes (%)				
NO	1277 (76.7)	213 (69.2)	1064 (78.5)	0.001**
YES	387 (23.3)	95 (30.8)	292 (21.5)	
COH protocol (%)				
Early follicular phase GnRHa long protocol	30 (1.8)	9 (2.9)	21 (1.5)	0.592
GnRHa long protocol	1208 (72.6)	224 (72.7)	984 (72.6)	
GnRH antagonist protocol	384 (23.1)	68 (22.1)	316 (23.3)	
luteal phase stimulation protocol	4 (0.2)	0 (0.0)	4 (0.3)	
PPOS protocol	17 (1.0)	3 (1.0)	14 (1.0)	
Ultra long GnRHa protocol	21 (1.3)	4 (1.3)	17 (1.3)	
Fertilization method (%)				
IVF	1286 (77.3)	240 (77.9)	1046 (77.1)	0.825
ICSI	378 (22.7)	68 (22.1)	310 (22.9)	
Endometrial preparation (%)				
modified natural cycles	552 (33.2)	95 (30.8)	457 (33.7)	0.063
mild stimulation	89 (5.3)	18 (5.8)	71 (5.2)	
HRT	754 (45.3)	130 (42.2)	624 (46.0)	
GnRHa_HRT	269 (16.2)	65 (21.1)	204 (15.0)	

*:p<0.05.

**:p<0.01.

***:p<0.001.

### Feature optimization

**Figure 2:**
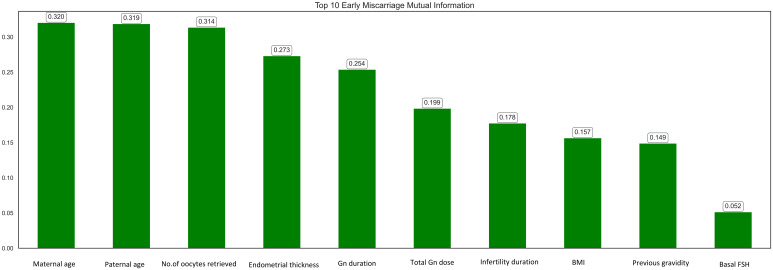
Top 10 features ranked by mutual information scores for predicting early miscarriage.

Mutual information (MI) analysis identified maternal age, paternal age, number of oocytes retrieved, and endometrial thickness as the top predictive features for early miscarriage (**Figure 2**). Other important features included Gn duration, total Gn dose, infertility duration, BMI, previous gravidity, and basal FSH. These findings align with clinical evidence, emphasizing the role of parental demographics, ovarian response, and endometrial conditions in determining pregnancy outcomes. The selected features were subsequently used for model development to enhance prediction accuracy.

Based on the recursive feature elimination (RFE) combined with a random forest classifier, the top 10 ranked features have been selected for the next step of model construction to predict early miscarriage. The selected features are maternal age, paternal age, BMI, basal FSH, basal LH, infertility duration, trigger day estradiol, total gn dose, number of oocytes retrieved, endometrial thickness.

Based on the results from mutual information analyses, and RFE analyses, the features selected for the next step of model construction are: maternal age, paternal age, previous gravidity, total Gn dose, number of oocytes retrieved, endometrial thickness, Gn duration, infertility duration, BMI, basal FSH, basal LH and trigger day estradiol.

### AUC analysis in training set

**Figure 3:**
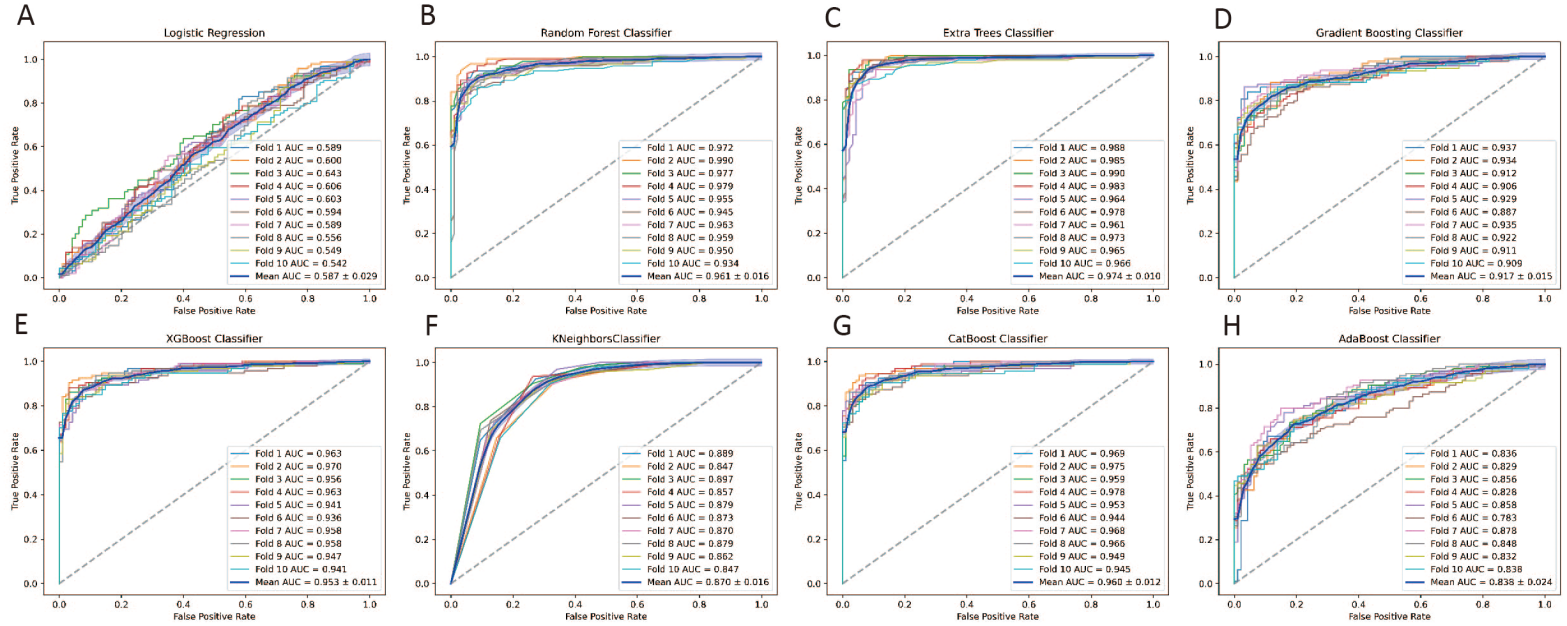
ROC curves Evaluating the performance of eight machine learning models with 10-Fold Cross-Validation on the Training Set for predicting early miscarriage. **(A)** Logistic Regression; **(B)** Random Forest classifier; **(C)** Extra Trees classifier;**(D)** Gradient Boosting classifier; **(E)** XGBoost classifier; **(F)** KNeighbors classifier; **(G)** CatBoost classifier; **(H)** AdaBoost classifier.

Receiver Operating Characteristic (ROC) analysis on the training set demonstrated superior performance of ensemble models compared to individual classifiers. Repeated 10-fold cross-validation confirmed the robustness of these results, ensuring reliable performance across different data subsets. Detailed comparisons of all models are provided in **Figure 3**.

### Models’ comparison in testing set

**Table 2** presents the average performance of these algorithms across six key metrics: Area Under the Curve (AUC), accuracy, recall, precision, F1-score, and specificity. Based on these metrics, the Gradient Boosting Classifier and the CatBoost Classifier emerged as the top-performing models. The Gradient Boosting Classifier leads in terms of AUC (0.831), accuracy (0.777), recall (0.649), and F1-score (0.744), demonstrating high effectiveness across various critical metrics. The CatBoost Classifier also exhibits strong performance, particularly in AUC (0.819), accuracy (0.754), precision (0.894), and specificity (0.932) (**Table 2**). These characteristics make both models robust and reliable for predicting early miscarriage following SVBT.

**Table 2:** Performance comparison of different machine learning models on the testing set.

Classifier	AUC	Accuracy	Recall	Precision	F1	Specificity
Logistic Regression	0.584	0.539	0.523	0.540	0.531	0.556
Random Forest Classifier	0.762	0.664	0.402	0.846	0.545	0.927
Extra Trees Classifier	0.717	0.587	0.210	0.856	0.337	0.965
Gradient Boosting Classifier	0.831	0.777	0.649	0.871	0.744	0.904
XGBoost Classifier	0.808	0.730	0.553	0.855	0.672	0.907
KNeighborsClassifier	0.603	0.573	0.563	0.575	0.569	0.583
CatBoost Classifier	0.819	0.754	0.576	0.894	0.700	0.932
AdaBoost Classifier	0.778	0.729	0.646	0.773	0.704	0.811
Voting Classifier*	0.836	0.780	0.619	0.914	0.738	0.942
Stacking Classifier*	0.823	0.769	0.619	0.884	0.728	0.919

*:Ensemble Model.

### Ensemble learning methods

**Figure 4:**
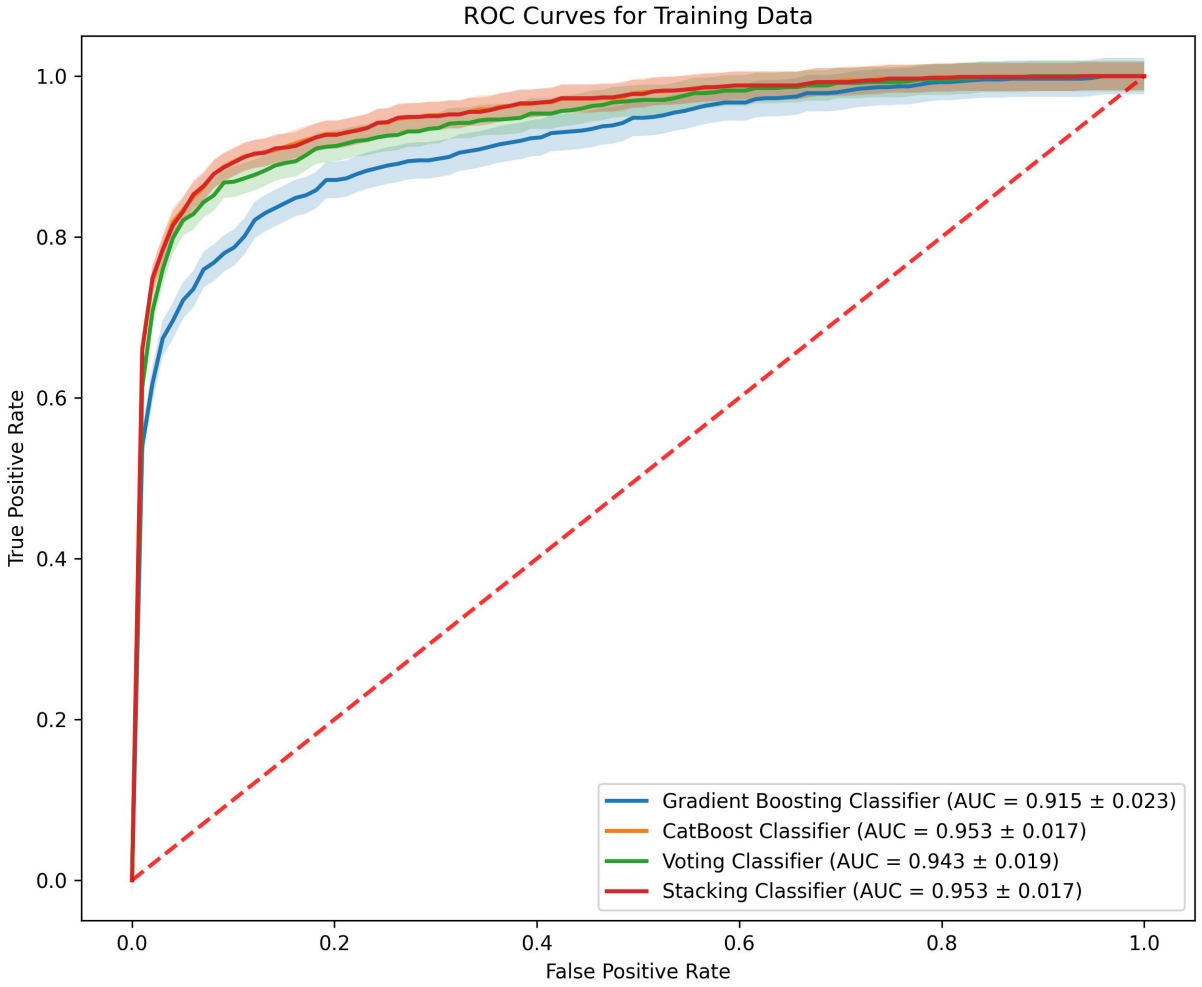
ROC curves comparing the performance of four machine learning models on the training data.

To enhance the performance of early miscarriage prediction models, this study employed ensemble learning methods by constructing two ensemble models based on the Gradient Boosting Classifier and the CatBoost Classifier: a Voting Classifier and a Stacking Classifier. In the training set, the ROC curves of the Voting Classifier and the Stacking Classifier demonstrate comparable performance to those of the individual Gradient Boosting Classifier and CatBoost Classifier (**Figure 4**).

In the evaluation of classifiers for predicting early miscarriage, the Voting Classifier demonstrated superior performance, leading in key metrics including AUC, where it scored 0.836, and accuracy, with a value of 0.780. This model also excelled in precision, achieving the highest among the classifiers at 0.914, and in specificity, where it led with a score of 0.942.

The Gradient Boosting Classifier also showed robust performance across various metrics. It ranked second in both AUC (0.831) and accuracy (0.777), indicating its strong capability to distinguish between cases. Additionally, it demonstrated good recall (0.649) and an F1 score (0.744), reflecting its balanced performance in identifying true positives while maintaining a lower rate of false negatives. The precision and specificity scores were also high at 0.871 and 0.904, respectively, reinforcing its applicability in diverse clinical environments where both sensitivity and precision are crucial (**Table 2**).

## Discussion

### Principal findings

The study demonstrates that ensemble machine learning models, particularly the Voting Classifier and Gradient Boosting Classifier, significantly improve the prediction of early miscarriage following SVBT cycles. The Voting Classifier achieved the highest performance metrics, with an AUC of 0.836, accuracy of 0.780, and precision of 0.914, underscoring its robustness and clinical applicability. The Gradient Boosting Classifier also exhibited strong predictive capability (AUC = 0.831, accuracy = 0.777), effectively capturing complex, non-linear interactions among features, such as parental age, endometrial thickness, ovarian response, and blastocyst quality. These findings underscore the effectiveness of ensemble methods in capturing the multifactorial nature of early miscarriage risk.

### Results in the context of what is known

Previous studies on early miscarriage prediction have primarily relied on traditional statistical models, such as logistic regression and LASSO regression, which have typically demonstrated only moderate predictive performance, with AUC values ranging from 0.615 to 0.660 (6). These methods often struggle to capture the complex, non-linear relationships among predictive variables, thereby limiting their accuracy and generalizability.

Ensemble learning models offer a promising alternative by integrating multiple algorithms, leading to improved predictive accuracy and robustness. Prior research has highlighted the advantages of ensemble methods in pregnancy-related predictions, including naturally conceived pregnancies (10). However, these models have not been specifically optimized for SVBT cycles, which involve unique physiological factors such as endometrial synchronization and blastocyst vitrification.

The superior performance of our proposed methodology can be attributed to several key factors. First, ensemble learning methods aggregate predictions from multiple models, reducing individual model biases and enhancing generalization. The Voting Classifier, in particular, leverages the strengths of multiple base models, producing more stable and accurate predictions. Second, Gradient Boosting enhances feature importance by iteratively improving weak learners, making it highly effective in handling the intricate dependencies among clinical and embryonic factors. Unlike traditional statistical models that assume linear relationships, boosting techniques dynamically refine decision boundaries, leading to superior classification performance.

Additionally, advanced feature selection techniques, including Mutual Information (MI) and Recursive Feature Elimination (RFE), were incorporated to improve model interpretability and efficiency. By systematically removing irrelevant or redundant features, our models focus on the most clinically meaningful predictors, such as maternal age, endometrial thickness, and embryo quality, thereby enhancing both predictive accuracy and generalizability.

This study addresses a critical gap by demonstrating that ensemble learning models significantly improve early miscarriage risk prediction in SVBT cycles, achieving higher accuracy and reliability compared to traditional approaches. These findings establish a new benchmark for predictive modeling in ART and highlight the potential of machine learning in enhancing personalized risk assessment and clinical decision-making.

### Clinical and research implications

The findings of this study hold significant implications for clinical practice. The enhanced predictive accuracy of ensemble machine learning models offers the potential for more personalized care in ART. Clinicians can leverage these models to identify pregnancies at high risk of early miscarriage, enabling closer monitoring and tailored counseling, while patients with lower risk might benefit from reduced interventions.

Integrating these machine learning models into electronic medical record (EMR) systems could further streamline risk assessment, providing real-time, data-driven support for clinical decision-making. Beyond their immediate clinical utility, these findings also pave the way for future research to investigate additional predictors of early miscarriage, including genetic, molecular, and lifestyle factors, to further refine and enhance model performance.

### Strengths and limitations

This study has several notable strengths. First, it is the first to develop ensemble learning models specifically designed for predicting early miscarriage in SVBT cycles, addressing a critical gap in the literature. Second, the study utilized a large and well-documented dataset from two reproductive medicine centers, which enhances both the reliability and generalizability of the findings. Third, the use of rigorous validation techniques, such as repeated stratified 10-fold cross-validation, ensured robust model performance and reduced the risk of overfitting.

Despite these strengths, several limitations should be acknowledged. The retrospective design may introduce biases related to data collection and patient selection. The absence of preimplantation genetic testing in the dataset restricts the ability to account for chromosomal abnormalities, a major contributor to early miscarriage. Furthermore, the dataset lacked sociodemographic information, such as socioeconomic status and education level, which are known to influence pregnancy outcomes. Lastly, while the models were specifically developed for SVBT cycles, their generalizability to other ART procedures or naturally conceived pregnancies remains to be validated in future studies.

## Conclusion

The study underscores the potential of ensemble machine learning models, particularly the Voting Classifier and Gradient Boosting Classifier, to significantly enhance the prediction of early miscarriage following SVBT. With the continued evolution of machine learning techniques, these models hold considerable promise in advancing clinical decision-making by delivering more accurate and personalized risk assessments.

## Data Availability

The datasets generated and/or analyzed during the current study are not publicly available due to the need for approval from relevant institutions or ethics committees. However, they are available from the corresponding author upon reasonable request and with appropriate approvals.

## References

[1] Quenby S Gallos ID Dhillon-Smith RK Podesek M Stephenson MD Fisher J. Miscarriage matters: the epidemiological, physical, psychological, and economic costs of early pregnancy loss. Lancet. (2021) 397:1658–67. doi: 10.1016/S0140-6736(21)00682-6 33915094

[2] Wang M Yang X Li L Zhu H Zhang H Jiang Y. Incidence and risk factors for early pregnancy loss in women with first pregnancy undergoing *in vitro* fertilization-embryo transfer. BMC Pregnancy Childbirth. (2022) 22:575. doi: 10.1186/s12884-022-04904-8 35854214 PMC9295353

[3] Emarah MSA El-Naggar MA Shabacy AE Qushwa SH. Hypothyroidism in women with recurrent spontaneous abortion. Adv Soc Sci Res J. (2020) 7:71–7. doi: 10.14738/assrj.710.9132

[4] Vomstein K Voss P Molnar K Ainsworth A Daniel V Strowitzki T. Two of a kind? Immunological and clinical risk factors differ between recurrent implantation failure and recurrent miscarriage. J Reprod Immunol. (2020) 141:103166. doi: 10.1016/j.jri.2020.103166 32623188

[5] Amitai T Kan-Tor Y Or Y Shoham Z Shofaro Y Richter D. Embryo classification beyond pregnancy: early prediction of first trimester miscarriage using machine learning. J Assist Reprod Genet. (2023) 40:309–22. doi: 10.1007/s10815-022-02619-5 PMC9935804 36194342

[6] Zhang M Ji X Hu X Zhu Y Ma H Xu H. Development and validation of a visualized prediction model for early miscarriage risk in patients undergoing IVF/ICSI procedures: A real-world multi-center study. Front Endocrinol. (2024) 14:1280145. doi: 10.3389/fendo.2023.1280145 PMC10905617 38433972

[7] Mahajan P Uddin S Hajati F Moni MA. Ensemble learning for disease prediction: A review. Healthcare. (2023) 11:1808. doi: 10.3390/healthcare11121808 37372925 PMC10298658

[8] Kabiraj S Raihan M Alvi N Afrin M Akter L Sohagi SA. Breast cancer risk prediction using XGBoost and random forest algorithm. In: 2020 11th International Conference on Computing, Communication and Networking Technologies (ICCCNT). IEEE, Kharagpur, India (2020). p. 1–4. doi: 10.1109/ICCCNT49239.2020.9225451

[9] Shalini M Radhika S. IG-ANGO: A novel ensemble learning algorithm for breast cancer prediction using genomic data. Evol Syst. (2024) 15:2399–418. doi: 10.1007/s12530-024-09619-z

[10] Lokhande A Gimovsky A Sarkar I. Predicting miscarriage and stillbirth using weighted ensemble machine learning [ID: 1338167. Obstet Gynecol. (2023) 141:28S–S. doi: 10.1097/01.AOG.0000930064.05901.88

[11] Singh S Tiwari S Goel P Tiwari DA. Retrospective: sightseeing excursion of threatened miscarriage pertaining ensemble machine learning algorithms. In: 2023 6th International Conference on Information Systems and Computer Networks (ISCON). IEEE, Mathura, India (2023). p. 1–7. doi: 10.1109/ISCON57294.2023.10111961

[12] Ueno S Ito M Uchiyama K Okimura T Yabuuchi A Kobayashi T. Closed embryo culture system improved embryological and clinical outcome for single vitrified-warmed blastocyst transfer: A single-center large cohort study. Reprod Biol. (2019) 19:139–44. doi: 10.1016/j.repbio.2019.03.004 30948345

[13] Khan I Khare BK. Exploring the potential of machine learning in gynecological care: A review. Arch Gynecol Obstet. (2024) 309:2347–65. doi: 10.1007/s00404-024-07479-1 38625543

[14] Mennickent D Rodríguez A Opazo M Riedel CA Castro E Eriz-Salinas A. Machine learning applied in maternal and fetal health: A narrative review focused on pregnancy diseases and complications. Front Endocrinol. (2023) 14:1130139. doi: 10.3389/fendo.2023.1130139 PMC10235786 37274341

[15] Islam MN Mustafina SN Mahmud T Khan NI. Machine learning to predict pregnancy outcomes: A systematic review, synthesizing framework and future research agenda. BMC Pregnancy Childbirth. (2022) 22:348. doi: 10.1186/s12884-022-04594-2 35546393 PMC9097057

[16] Peluso C Oliveira RD Laporta GZ Christofolini DM Fonseca FLA Laganà AS. Are ovarian reserve tests reliable in predicting ovarian response? Results from a prospective, cross-sectional, single-center analysis. Gynecol Endocrinol. (2021) 37:358–66. doi: 10.1080/09513590.2020.1786509 32613875

[17] Gardner DK Schoolcraft WB. Culture and transfer of human blastocysts. Curr Opin Obstet Gynaecol. (1999) 11:307–11. doi: 10.1097/00001703-199906000-00013 10369209

[18] Cozzolino M Vitagliano A Di Giovanni MV Laganà AS Vitale SG Blaganje M. Ultrasound-guided embryo transfer: summary of the evidence and new perspectives. A systematic review and meta-analysis. Reprod Biomed Online. (2018) 36:524–42. doi: 10.1016/j.rbmo.2018.01.015 29576332

[19] Alkhawaldeh IM Albalkhi I Naswhan AJ. Challenges and limitations of synthetic minority oversampling techniques in machine learning. World J Methodol. (2023) 13:373–8. doi: 10.5662/wjm.v13.i5.373 PMC10789107 38229946

